# Subsequent mammography reduces recall and increases breast cancer detection: an audit of a screening program

**DOI:** 10.61622/rbgo/2025rbgo89

**Published:** 2025-11-18

**Authors:** Erika Marina Solla Negrao, Mariana Alves Almeida, Emanuele Françoso Cardoso, Alvaro Silva Almeida, Samanta Santos Sousa, Cassio Cardoso-Filho, Rodrigo Menezes Jales, Diama Bhadra Vale

**Affiliations:** 1 Universidade Estadual de Campinas Faculdade de Ciências Médicas Department of de Tocogynecology Campinas SP Brazil Department of de Tocogynecology, Faculdade de Ciências Médicas, Universidade Estadual de Campinas, Campinas, SP, Brazil.; 2 Hospital de Câncer de Barretos Instituto de Prevenção de Câncer Campinas SP Brazil Instituto de Prevenção de Câncer, Hospital de Câncer de Barretos, Campinas, SP, Brazil.

**Keywords:** Breast neoplasms, Quality of health care, Screening, Early detection of cancer, Mammography, Breast density, Biopsy, Prevalence

## Abstract

**Objective:**

To analyze recall rates in a public breast cancer screening facility in Campinas, Brazil.

**Methods:**

A prospective assessment of outcomes on screening mammographies (MMG) between July 2023 and August 2024. BI-RADS® 0,4/5 indicated positive results, and women recalled. The variables were age, whether first or subsequent MMG, and biopsy (cancer positive or negative). The outcomes were recall rate and cancer detection rate on the recall (CDR). Prevalence ratio with 95% confidence interval (PR) estimated the risk.

**Results:**

There were included 19,377 MMG on women over 40: 15,983 subsequent MMG (82.5%), and 1,646 women recalled (BR 0,4/5). Adherence to recall was over 99%. The recall rates were 12.4% at first and 7.7% at subsequent MMG. Recall rate was 1.6 times higher at first than at subsequent MMG (PR 1.61;1.45-1.79). CDR was higher at subsequent MMG. A first MMG reduced the risk of cancer detection by 71% (PR 0.29-0.15;0.58). Compared to women 50-69, there were no differences in the risks of recall and cancer detection at first MMG. At subsequent MMG the recall risk was higher in women 40-49 (PR 1.16;1.03-1.30), and over 69 (PR 1.47;1.03-2.12). The risk of cancer detection was 60% lower in women 40-49 (PR 0.60;0.36-0.99), and 2.7 times higher in women over 69 (PR 2.78;1.32-5.84).

**Conclusion:**

The recall rates were 12.4% at first and 7.7% at subsequent MMG. Adherence was high. Screening efficiency was higher in women 50-69. At subsequent screenings women 40-49 showed a higher recall rate and a lower CDR when compared to women 50-69.

## Introduction

The recall rate is an important quality indicator used to assess the performance of breast cancer screening and early detection programs that rely on mammography. It refers to the proportion of individuals who are asked to return for additional diagnostic procedures following a screening mammography that shows potentially abnormal or inconclusive findings. An appropriate recall rate reflects a balance between detecting as many true cases of cancer as possible and minimizing unnecessary follow-up tests for false-positive results. Recall rates vary significantly between countries, services, and among professionals who perform and analyze the scans.^(1–3)^ This variability can have significant clinical and economic implications, such as carrying out unnecessary procedures and follow-ups, additional costs for the health systems, and the distress associated with false-positives.^(4)^ Furthermore, the evidence linking recall and cancer detection rates is conflicting.^(5)^

It is important that healthcare providers and administrators are aware of and acknowledge the factors that contribute to and influence recall rates. This is particularly critical in the context of false positives, in order to mitigate the potential harms associated with inaccurate results. In its recommendations regarding benchmarks and acceptable ranges for mammographic screening, the Breast Imaging Reporting and Data System (BI-RADS^®^) defines the reference value for the abnormal interpretation rate (i.e., recall rate) as 10.6%, with an acceptable range between 5% and 12%.^(6)^

The optimal point of balance - where the maximum benefit is achieved with minimal drawbacks ideal range in digital mammography appears to lie between 7% and 9%.^(3)^ In this context, it describes the range in which nearly all cancer cases are identified while the number of false positives remains low.^(3)^

Several factors are known to be associated with an increased likelihood of patient recall, including: age under 50 years, a history of previous abnormal findings, an annual radiologist volume of fewer than 1,250 exams, and fewer than 10 years of radiologist experience.^(7)^

In Brazil, mammography screening is recommended for women aged 40 and over by some medical organizations; however, these examinations are often performed opportunistically, without a structured population-based invitation program for eligible women.^(8,9)^ There is also a divergence in national guidelines: while some medical societies advocate for annual screening starting at age 40, the Brazilian National Cancer Institute (INCA) recommends biennial screening for women aged 50 to 69 years. In addition, national publications or statistics on recall rates are scarce within Brazil. A descriptive study from 2018 based on the national information system included results from women aged 50 to 69 years who had had mammograms in settings showing BI-RADS^®^2 rates higher than 75% and BI-RADS^®^0 rates lower than 12%.^(10)^ The overall rate of positive mammograms was 9.0%, but regional studies with a broader inclusion criteria point to the rates being above 10%.^(11,12)^ This variation in rates indicates a need for standardization to use the actual positive rate in quality control assessments of healthcare services and of the performance of screening programs.

This study evaluated a Brazilian population-based public facility for breast cancer screening to identify and analyze recall rates. Our aim was to generate evidence to support national recommendations and contribute high-quality data to the international literature regarding the performance of a Brazilian screening program.

## Methods

A prospective audit of the results, adherence, and outcomes of further assessment on screening mammographies performed between July 2023 and August 2024 in Campinas, São Paulo, Brazil. The public health system of the city recommends annual mammographies for women aged 40 to 49 and biennially for women aged 50 to 69. The total female population aged 40 to 69 in Campinas was 232,866 in 2022,^(13)^ and it is estimated that in that year 48% of these women (121,464) were exclusive users of the public health system.^(14)^

The mammographies were performed at the "Cancer Prevention Institute," a satellite unit of the "Barretos Cancer Hospital," in São Paulo state. The Institute is the referral unit for breast cancer screening and diagnosis in Campinas, where screening mammographies are performed on behalf of the public health system. Two other units also perform diagnostic mammographies. However, they are hospital-based and screen mainly women treated within other health problems, including breast cancer survivors. Primary health care units refer women who undergo mammography to the Institute, but they can also elect to undergo the exam under their own volition, as long as they are within the recommended age group and test intervals. Some exams are performed in one mobile unit linked to the central unit, facilitating access to the general public. The data from the mobile unit was included in the study.

Quality control guidelines on positioning and radiation usage set by the national bodies already exist for digital mammography equipment.^(15)^ The exams are read twice, once by a trained, experienced and, certified radiologist and once by an specialist Aritificial Intelligence software. When the results between the readings differ, a senior radiologist performs a third reading. The reading team remained unchanged during the study period. The results from the reading team followed the nomenclature of the BI-RADS^®^ classification from 0 to 5.^(16)^ Breast density was determined by the radiologist's visual assessment of the density and adipose composition of the tissue.^(16)^ Data was also recorded about the patients’ entire interaction with the unit using digital record keeping.

The study included all women over 40 years old who underwent mammography during the study period. Women who were symptomatic and had undergone diagnostic mammography were excluded from the study. Sociodemographic and clinical information, such as cancer family history, was not accessed.

A BI-RADS^®^ report of 0, 4, and 5 indicates a positive result, and the patient was then recalled. While there is no system for calling the entire target population (opportunistic screening), positive cases are individually re-called by the unit. Women with a BI-RADS® 0 result were referred to ultrasound and, if necessary, a different mammography incidence, and then re-categorized to a new BI-RADS® result. All BI-RADS® 4 and 5 were then submitted to a biopsy, preferably by ultrasound. However, they can also be performed by mammography (stereotaxis) when this method exclusively visualizes the area of interest. Further assessments of positive results are performed by the same radiological team, aided by a breast surgeon when surgical biopsies are needed. A central referral laboratory examines the anatomicopathological samples using immunohistochemistry. The breast surgeon evaluates all cancer cases for the initial staging with the complementary exams required and then refers them to an appropriate treatment unit.

The variables of interest were patient age, the reason for the mammography (screening or diagnostic), whether it was the first or a subsequent mammography, the initial mammogram result, the results of any further assessments (mammography and/or ultrasound), and any biopsy results. The anatomicopathological results were categorized as positive or negative for cancer, and carcinomas *in situ* were grouped with invasive carcinomas to indicate positive cases of cancer. Only screening mammographies performed on women over 40 years of age were included in this study.

The primary outcome was the recall rate, calculated by the number of positive tests (BR0, 4/5) as the numerator, and the total number of tests by age group as the denominator. The rates for each BI-RADS^®^ result were also calculated. The secondary outcome was the cancer detection rate, calculated by the number of positive cancer results as the numerator and the total number of positive tests by age group, including non-adherent cases (0.1% with the first screening and 0.06% subsequently), as the denominator. The Prevalence Ratio was calculated to estimate the risk, considering a 95% Confidence Interval. The significance level admitted was 5%. Data were stratified by age, and no other adjustment was made. We used the SAS System for Windows (Statistical Analysis System), version 9.4 (SAS Institute Inc, 2002-2012, Cary, NC, USA).

The study was approved by the Ethics Committee of Unicamp under CAAE number: 60402322.7.1001.5404 (approval number 5.600.419). The Committee waived the requirement to confirm patient consent, considering the audited nature of the data.

## Results

**Figure 1:**
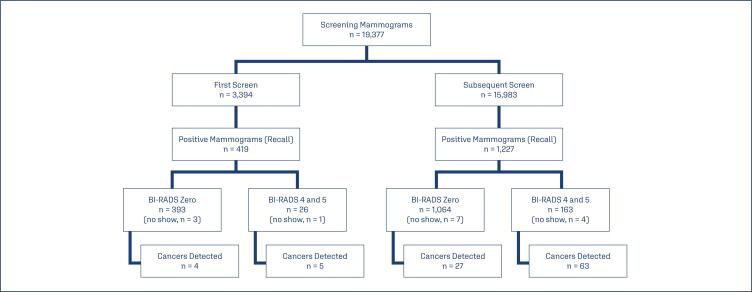
Flowchart of the study population from screening to recall and cancer diagnosis

A total of 19,377 mammographies done on women aged between 40 and 94 performed between July 2023 and August 2024 were included in this study. The flowchart of the study population, from screening to recall and cancer diagnosis, is shown in figure 1. Exams conducted among women aged between 40 and 49 accounted for 35.7% of cases, while women from 50 to 69 accounted for 62.9% of cases. In total, 15,983 of the women had previously undergone a mammogram (82.5%). There were 1,646 positive cases (BR 0, 4/5), of which 419 were first exams and 1,227 were subsequent exams. The classification of the results was Bi-RADS®0 in 1,457 exams, BI-RADS^®^ 4 in 183 exams, and BI-RADS^®^ 5 in 6 exams. The radiological pattern of the breast was classified as dense in 70.5% of cases and adipose in 29.5% of cases (Table 1).

**Table 1:** Descriptive analysis of screening mammographies

	Screen		Total n(%)
	First n(%)	Subsequent n(%)	
Total mammograms	3,394	15,983	19,377
40 to 49 years	2,497(73.6)	4,428(27.7)	6,925(35.7)
50 to 69 years	876(25.8)	11.304(70.7)	12,180(62.9)
70 years or older	21(0.6)	251(1.6)	272(1.4)
Positive mammograms	419	1,227	1,646
BI-RADS^®^ 0	393(93.8)	1,064(86.7)	1,457(88.5)
BI-RADS^®^ 4 and 5	26(6.2)	163(13.3)	189(11.5)
Radiological density on positives			
Dense	314(74.9)	846(68.9)	1,160(70.5)
Not dense	105(25.1)	381(31.1)	486(29.5)

A total of 199 mammographies and 1,568 ultrasounds were performed as recalled cases, but an additional 15 women did not attend when recalled (99.1% adherence). A total of 172 mammography and 48 ultrasound biopsies were performed (Table 2).

**Table 2:** Descriptive analysis of further assessment in positive mammographies

	First mammography result	
	BI-RADS^®^ 0	BI-RADS^®^ 4/5
Biopsy by mammography	2	133
Biopsy by ultrasound	46	39
Total*	48	172

* Some information whether biopsy by mammography or ultrasound is missing.

The recall rates were 12.4% when the patient underwent their first mammography and 7.7% for patients undergoing a subsequent exam (Table 3). At the first exam, the recall rate was 1.6x higher than for subsequent exams (PR 1.61; 95% CI 1.45-1.79), being 1.5x higher in both women aged 40 to 49 (PR 1.51; 95% CI 1.31-1.74) and 50 to 59 (PR 1.52; 95% CI 1.24-1.85). Cancer detection rates in these recall exams were higher for those undergoing a subsequent examination. Performing the exam for the first time reduced the risk of having a cancer detected by 71% in the recall examination (PR 0.29; 95% CI 0.15-0.58).

**Table 3:** Analysis of recall rates and cancer detection rates in screening mammographies in Campinas, Brazil, regarding first or subsequent screen

	First screen n/d(%) (Recall Rate)	Subsequent screen n/d(%) (Recall Rate)	PR of Recall (95% CI)	p-value
40 to 49 y	319/2,497(12.8)	375/4,428(8.5)	1.51 (1.31;1.74)	<0.001
50 to 69 y	97/876(11.1)	825/11,304(7.3)	1.52 (1.24;1.85)	<0.001
+69 y	3/21(14.3)	27/251(10.7)	1.33 (0.44;4.02)	0.713
Total	419/3,394(12.4)	1.227/15,983(7.7)	1.61 (1.45;1.79)	<0.001
	**First screen** **n/d (CDR)**	**Subsequent screen** **n/d (CDR)**	**PR of CDR** **(95% CI)**	pvalue
40 to 49 y	5/319(1.6)	18/375 (4.8)	0.33 (0.12;0.87)	0.018
50 to 69 y	4/97(4.1)	66/825(8.0)	0.52 (0.19;1.38)	0.173
+69 y	0/3(-)	6/27(22.2)	0.54 (0.04;7.84)	1.000
Total	9/419(2.2)	90/1,227(7.3)	0.29 (0.15;0.58)	<0.001

y – years; PR – Prevalence Ratio; CI – Confidence Interval; CDR – Cancer Detection Rate

When comparing the age groups, with women aged 50 to 69 as the reference group, there were no differences in the risks of recall and cancer detection in women who performed the exam for the first time. Among women who underwent subsequent exams, the risk of recall was higher in women aged 40 to 49 years (PR 1.16; 95% CI 1.03-1.30), and for those over 69 (PR 1.47; 95% CI 1.03-2.12). The risk of cancer detection was 60% lower in women aged 40 to 49 (PR 0.60; 95% CI 0.36-0.99), and 2.7x higher in women over 69 years (PR 2.78; 95% CI 1.32-5.84) (Table 4).

**Table 4:** Analysis of recall rates and cancer detection rates in screening mammographies in Campinas, Brazil, regarding age group

	Recall rate					
	First screen			Subsequent screen		
	n/d	PR (95% IC)	p-value	n/d	PR (95% CI)	p-value
40 to 49 y	319/2,497	1.15 (0.93;1.43)	0.187	375/4,428	1.16 (1.03;1.30)	0.013
50 to 69 y	97/876	Reference		825/11,304	Reference	
+69 y	3/21	1.29 (0.45;3.74)	0.721	27/251	1.47 (1.03;2.12)	0.038
	**Cancer detection rate**					
	**First screen**			**Subsequent screen**		
	**n/d**	**PR (95% IC)**	**p-value**	**n/d**	**PR (95% CI)**	**p-value**
40 to 49 y	5/319	0.38 (0.10;1.39)	0.223	18/375	0.60 (0.36;0.99)	0.044
50 to 69 y	4/97	Reference		66/825	Reference	
+69 y	0/3	2.72 (0.17;42.39)	1.000	6/27	2.78 (1.32;5.84)	0.009

y – years; n – absolute number; d – denominator; PR – Prevalence Ratio; CI – Confidence Interval

## Discussion

In this audited analysis of the performance of mammographic screening for breast cancer in Campinas, a densely populated city in Brazil, we observed recall rates of 12.4% with the first exam and 7.7% with subsequent exams. These rates were higher with women screened outside the 50 to 69 age group. Cancer detection in the recall subsequent mammography was 7.3%, and was 40% lower in women aged 40 to 49 compared to women aged 50 to 69.

The frequency of Bi-RADS^®^0 in this sample was 12.0% in the first screening and 6.8% in the subsequent exams, with a frequency of Bi-RADS^®^4 and 5 of approximately 1%. Recall rates were therefore higher from the first exam compared to the subsequent ones. These proportions are considered acceptable by the international literature, but conclusions are not unanimous. One study suggested that the ideal recall point is between 12% and 14% and indicated any less than 10% may be too low.^(1)^ In another study, the ideal point appeared to be between 7% and 9% for 2D and 3D digital mammography.^(3)^ Other authors have found that the availability of previous mammographies reduces recall, false positive, and abnormal interpretation rates.^(17,18)^ In the UK Age Trial Study, the false-positive mammography rates in the first and subsequent routine screenings were 4.9% and 3.2%, respectively.^(2)^

Recall is an effective strategy for stratifying risks and highlighting the clinical value of a negative mammogram. However, increasing recall rates does not necessarily imply an increase in the proportion of cancer detection; instead, it may lead to an increase in the proportion of false-positive results.^(2,4)^ Furthermore, the possibility of overdiagnosis and the anxiety related to false-positive results are known factors indicated as harms of screening.^(8–19)^ The overuse of the program without a corresponding increase in effectiveness can be challenging in low- and/or middle-income scenarios and may lead to harm to the population.

In our sample, cancer detection rates from the recall exams were higher when it was a subsequent mammography. Performing the exam for the first time reduced the risk of having cancer detected by 71% when recalled (PR 0.29; 95% CI 0.15-0.58). When comparing other age groups to those between 50 and 69, there were no differences in the risks of recall and cancer detection in women who had undergone the exam for the first time.

Adherence to recall was 99%. Authors describe that in practice, access to exams depends on the structure of health services in the region, and many women do not have the possibility of being screened regularly or may not get adequate further assessment.^(8,20)^ In Campinas, the screening team actively invites positive cases using a phone call or text message. Studies have shown that personal invitations like these to remind patients of appointments result in high rates of adherence.^(21)^

The proportion of women aged 40 to 49 who were screened in our study was 35.7%, which contributed to the high proportion of dense breasts (71%). Among the women who underwent a subsequent examination, the recall risk was 1.2 times higher in this age group, and the cancer detection rate was 60% lower. An annual mammography before the age of 50 is associated with a relative reduction in breast cancer mortality, which was attenuated after 10 years, although the absolute reduction remained constant.^(22)^

There is a lot of criticism regarding the screening of women under 50, with the main argument being that the low efficiency of this strategy increases the false-positive results and overscreening rates.^(23)^ In this study, we observed an elevated proportion of women under 50 being screened. Also, when compared to women aged 50 to 69, the target group recommended by the Ministry of Health,^(8)^ a higher rate of recall and a lower rate of cancer detection was found. In Campinas and most Brazilian municipalities, screening is extensively performed targeting women under 50, following the recommendations of various medical societies.^(9)^ In fact, these divergencies about whether to screen women under 50 or not hide the fact that the coverage of women from 50 to 69 is low, and overall, women with positive results face many barriers to getting further assessment nationwide.

From the subsequent screens, only 11% of women over 69 years old were positive, but 22% of the recalled cases were cancer cases. A possible explanation would be that this population voluntarily sought screening due to possible personal or family risk factors or even due to previous diagnostic investigations. There has been a recent increase in organizations recommending screening up to 74 years of age.^(19,24)^ This discussion is currently underway, weighing up the possible benefits of increased detection against the risks of overdiagnosis.^(25)^

The strength of this study was the evaluation of data from an organized screening service with an extremely high adherence of recalled cases and further assessment, uncommon in low- and middle-income regions. The facility is a referral center for women at normal risk of breast cancer in a populous city in Brazil, reflecting the population living in similar areas.

The main limitation is the short observation time and the failure to account for interval cancers. A cohort with the evolution of cases is ongoing. The single-center design limits generalizability, making a more robust analysis difficult due to the low number of cases and lack of comparison with different services. Finally, the absence of data on socioeconomic status and family history limits the evaluation of the screening outcomes.

## Conclusion

Screening efficiency was highest among women aged 50 to 69, who also represented the most frequent age group in the sample. Importantly, subsequent mammograms demonstrated superior performance, with lower recall rates and higher cancer detection rates compared to first-time screenings. In contrast, women aged 40 to 49 undergoing subsequent exams exhibited higher recall rates and lower cancer detection rates than those aged 50 to 69. These findings reinforce the central role of subsequent mammograms in improving screening efficiency and support prioritizing the 50 to 69 age group in population-based screening policies. They also highlight the importance of organized invitation systems to promote adherence. Finally, the results underscore the need for further evaluation of the cost-effectiveness and clinical value of extending routine screening to women in their 40's within the Brazilian healthcare context.
